# Superior semicircular canal dehiscence and subsequent closure induces reversible impaired decision-making

**DOI:** 10.3389/fneur.2023.1259030

**Published:** 2023-10-12

**Authors:** Todd M. Mowery, P. Ashley Wackym, Jacqueline Nacipucha, Evelynne Dangcil, Ryan D. Stadler, Aaron Tucker, Nicolas L. Carayannopoulos, Mina A. Beshy, Sean S. Hong, Justin D. Yao

**Affiliations:** ^1^Department of Otolaryngology – Head and Neck Surgery, Rutgers Robert Wood Johnson Medical School, New Brunswick, NJ, United States; ^2^Rutgers Brain Health Institute, New Brunswick, NJ, United States

**Keywords:** cognitive dysfunction, decision-making, dizziness, headache, migraine, superior semicircular canal dehiscence, vestibular

## Abstract

**Background:**

Vestibular loss and dysfunction has been associated with cognitive deficits, decreased spatial navigation, spatial memory, visuospatial ability, attention, executive function, and processing speed among others. Superior semicircular canal dehiscence (SSCD) is a vestibular-cochlear disorder in humans in which a pathological third mobile window of the otic capsule creates changes to the flow of sound pressure energy through the perilymph/endolymph. The primary symptoms include sound-induced dizziness/vertigo, inner ear conductive hearing loss, autophony, headaches, and visual problems; however, individuals also experience measurable deficits in basic decision-making, short-term memory, concentration, spatial cognition, and depression. These suggest central mechanisms of impairment are associated with vestibular disorders; therefore, we directly tested this hypothesis using both an auditory and visual decision-making task of varying difficulty levels in our model of SSCD.

**Methods:**

Adult Mongolian gerbils (*n* = 33) were trained on one of four versions of a Go-NoGo stimulus presentation rate discrimination task that included standard (“easy”) or more difficult (“hard”) auditory and visual stimuli. After 10 days of training, preoperative ABR and c+VEMP testing was followed by a surgical fenestration of the left superior semicircular canal. Animals with persistent circling or head tilt were excluded to minimize effects from acute vestibular injury. Testing recommenced at postoperative day 5 and continued through postoperative day 15 at which point final ABR and c+VEMP testing was carried out.

**Results:**

Behavioral data (d-primes) were compared between preoperative performance (training day 8–10) and postoperative days 6–8 and 13–15. Behavioral performance was measured during the peak of SSCD induced ABR and c + VEMP impairment and the return towards baseline as the dehiscence began to resurface by osteoneogenesis. There were significant differences in behavioral performance (d-prime) and its behavioral components (Hits, Misses, False Alarms, and Correct Rejections). These changes were highly correlated with persistent deficits in c + VEMPs at the end of training (postoperative day 15). The controls demonstrated additional learning post procedure that was absent in the SSCD group.

**Conclusion:**

These results suggest that aberrant asymmetric vestibular output results in decision-making impairments in these discrimination tasks and could be associated with the other cognitive impairments resulting from vestibular dysfunction.

## Introduction

In adults, vestibular loss and dysfunction has been associated with cognitive deficits. Specifically, vestibular loss in adults is associated with decreased spatial navigation (1), spatial memory (2), visuospatial ability (3–6), attention (7, 8), executive function (5, 9), and processing speed (5), among others. In addition, individuals with chronic vestibular disorders have been reported to have accompanying cognitive dysfunction (9–13). These cognitive impairments can be observed in patients within a group of vestibular-cochlear disorders referred to as “Third Window Syndrome.” In this disorder a pathological third mobile window of the otic capsule creates changes to the flow of sound pressure energy between the oval and round window (see review 14). The nature and location of this third mobile window can occur at many different sites (or multiple sites); however, the most common third mobile window is superior semicircular canal dehiscence (SSCD), (9, 13, 15–18). The primary physiological symptoms include sound-induced dizziness/vertigo, inner ear/otic capsule conductive hearing loss, autophony (hearing internal sounds abnormally well [one-third can hear their eyes move or blink]), headaches, and visual problems (convergence disorders, skew deviation, nystagmus, oscillopsia). However, individuals also experience measurable deficits in basic decision-making, short-term memory, concentration, spatial cognition, and depression (9, 13). This can lead to significant decreases in quality of life, lower academic performance in children, and decreased job performance in adults. Even with surgical treatment (dehiscence plugging/resurfacing) these chronic symptoms can be persistent, and recovery can be prolonged. This suggests that central networks undergo maladaptive neural plasticity; however, there has been limited investigation into the vast vestibular projections that integrate with virtually every major cognitive behavioral and sensory system of the brain.

It has been suggested that the vestibular system can be considered a potential window for exploring brain function beyond that of maintenance of balance, and into areas of cognitive, affective and psychiatric symptomology (19). It is known that normal vestibular activity is important for accurate sound localization (20) and higher-order sensorimotor integration at the level of the cortex (21). Indeed, emerging research is also demonstrating that disruption of vestibular input can cause deficits to visuospatial processing, memory, navigation, attention, and executive function (7). To this end, neuropsychology studies before and after surgical management of third window syndrome, including SSCD, have shown that cognitive dysfunction can occur and improve over time (9). We have developed a gerbil model that is accompanied by peripheral measures of impairment (elevated ABR thresholds, increased c+VEMPs amplitudes) that manifest and then resolve as the surgically created SSCD closes by spontaneous osteoneogenesis; resulting in resurfacing of the canal (22). The current study’s scientific premise is that by utilizing this experimental model of SSCD we can design experiments that directly investigate the SSCD-induced changes to central plasticity along vestibular and auditory circuits that are associated with cognitive impairments, which then resolve after natural bone regrowth. This approach will allow us to explore the interactions between the vestibular nucleus and the vast integration of their long-range projections to the auditory system through neural recordings taken during behavioral paradigms that test higher order cognition such as decision-making. We found that animals were impaired on both an auditory and visual decision-making task shortly after SSCD, which corresponded with the peak of peripheral impairment that we previously reported (22). As these physiological symptoms return towards baseline, we find a reduction in the impairment; however, inter-animal variability in recovery rates shows that the vestibular component (c+VEMP amplitude) is directly correlated with behavioral impairment and recovery to preoperative levels. Together these results show that our model allows a unique timeline to investigate central cognitive components of vestibular induced impairment and recovery.

## Materials and methods

### Animals

A total of (33) adult male and female Mongolian gerbils *Meriones unguiculatus* met the inclusion criteria and were used in this study. All animals were housed in the same vivarium facility under a 12/12 dark cycle with *ad libitum* access to food and water. Surgical creation of a 1.5 mm fenestration of the superior (anterior) semicircular canal produced the SSCD. The details of this procedure have been published previously (22). Exclusion criteria included removing any animals with persistent circling or head tilt present at post SSCD day 3 from the study protocol. The Rutgers University IACUC reviewed and approved this research protocol (PROTO202000179).

### Auditory brainstem response testing

Animals were anesthetized with isoflurane (1.0%) and placed in a small sound chamber (IAC, Sound Room Solutions, Inc., Glen Cove, NY). Auditory brainstem response (ABR) recordings were made by inserting pin electrodes subcutaneously at the vertex of the skull and just caudal to the right pinna; the ground electrode was inserted into the base of the tail. BioSigRZ software and the TDT ABR system (Tucker-Davis Technologies, Alachua, FL, United States) were used to collect ABR data. A 10-cm tube (closed field) was inserted into the ear and placed at the opening of the ear canal. The left ear of the animal was stimulated *via* multi-field speaker (MF1, Tucker-Davis Technologies) at 1, 2, 4, 8, and 16 kHz tones (90 to 20 dB SPL [10 dB steps]), 5 ms, 2 ms linear ramp rise-fall times at 25 Hz. Traces were averaged across 500 (threshold) sweeps. Thresholds for each frequency were measured as the last dB SPL, i.e., 10 dB SPL resolution stimulus level, that elicited a tone-induced ABR.

### Sound-induced cervical positive potential vestibular evoked myogenic potentials

Sound-induced otolithic stimulation and evoked intramuscular excitatory potential recordings were made by inserting pin electrodes into the neck extensor muscles (splenius capitus m.) and the reference electrode in the vertex of the skull measured (positive cervical vestibular evoked potential [c+VEMP]). BioSigRZ software and the TDT ABR system were used to collect c+VEMP data. A 10-cm tube capable of delivering 100 dB SPL (see TDT specs, Closed Field) was inserted into the ear and placed at the opening of the ear canal. The left ear of the animal was stimulated via multi-field speaker (MF1, Tucker-Davis Technologies) at 2 kHz (100 to 80 dB SPL [5 dB steps], 5 ms, 2 ms linear ramp rise-fall times sampled at 25 kHz). Traces were averaged across 500 (threshold) sweeps. The c+VEMPs were recorded under low-isoflurane anesthesia (<1.5%), near conditions of wakefulness. The c+VEMP was measured when it appeared under the condition of stimulation of air-conducted sound at 2 kHz and 100 dB. Peak amplitudes were measured by subtracting the peak of the negative N1 wave (in μV) from the later positive P1 wave.

### Auditory discrimination task

We assessed auditory perceptual skill in gerbils with a positive reinforcement “Go-NoGo” appetitive conditioning paradigm. Briefly, gerbils were placed on controlled access to food and trained to discriminate between amplitude-modulated (AM) broadband noise presented at 4 versus 12 Hz. Gerbils were placed in a behavioral arena test cage housed in a sound attenuation chamber (Med Associates, Inc., Fairfax, VT) and observed via a closed-circuit monitor. Auditory stimuli were presented from a calibrated multifield speaker (MF1, Tucker-Davis Technologies, Alachua, FL, United States) positioned 15 inches above the test cage floor. Sound calibration measurements were made with a 1/4-inch free-field condenser recording microphone (Brüel & Kjær, Nærum, Denmark). A modular pellet dispenser (Med Associates, Inc., [20 mg]) was connected to a trough type pellet receptacle (Med Associates, Inc.) placed within the test cage, and a cylindrical nose port with a 1-inch diameter hole (Med Associates, Inc.) was placed on the opposite side. Sensitive infrared sensors bisected the nose port and pellet receptacles to detect gerbil nose and head entry, respectively. Stimuli, food reward delivery, and behavioral data acquisition were controlled by an iPac computer system running iCon behavioral interfaces (Tucker-Davis Technologies). Gerbils self-initiated trials by placing their nose in the noseport. On each trial, one of two stimulus types were presented. The “Go” stimulus consisted of AM broadband noise (25 dB roll-off at 3.5 and 20 kHz) with a modulation rate of 4 Hz and a modulation depth of 100%. The “NoGo” stimulus for the less difficult (“easy”) auditory discrimination task consisted of AM broadband noise presented at a modulation rate of 12 Hz at 100% modulation depth. The NoGo stimulus for the more difficult (“hard”) auditory discrimination task consisted of AM broadband noise presented at a modulation rate of 6 Hz at 100% modulation depth. All stimuli were presented at a sound level of 70 dB SPL and was recalibrated daily with a sound level meter. Gerbils were shaped to approach the food troughs upon presentation of the Go stimulus, and received a 20-mg pellet reward. Once gerbils reached a criterion of three consecutive days of 100 trials with >90% Hit (correctly approaching the food trough during a Go trial), they were then trained to repoke upon presentation of the NoGo stimulus. During this phase, NoGo trials (20% probability) were randomly interleaved with Go trials. Gerbils performed the task for at least 120 trials per day, or until over 20 NoGo trials occurred. Typically, a session lasted 45 min to 1 h. This training continued for 10 days.

Trials were scored as Hit, Miss (failing to approach the food trough and repoking during a Go trial), Correct Reject (CR; correctly repoking during a NoGo trial), or False Alarm (FA; incorrectly approaching the food trough on a NoGo trial). Hit and FA rates were constrained to floor (0.05) and ceiling (0.95) values. A performance metric, d-prime was calculated for each session by performing a *z*-transform of both hit rate and false alarm rate: d-prime = *z* (Hit rate) − *z* (FA rate). Criterion was set at d-primes of 1.5. It typically took normal hearing animals 3–4 days to achieve the criterion of d-prime 1.5 when performing the easy auditory discrimination task. It typically takes normal hearing animals 5–6 days to achieve the criterion of d-prime 1.5 when performing the hard auditory discrimination ask.

### Visual discrimination task

Visual perceptual skill was assessed with positive reinforcement “Go-NoGo” appetitive conditioning paradigm, similar to that as described for the AM rate auditory discrimination task above. In contrast to the AM rate auditory discrimination task, visual stimuli were delivered from a light-emitting diode (LED; Med Associates, Inc.). The Go stimulus consisted of a flashing LED presented at 4 Hz. The NoGo stimulus for the less difficult (“easy”) visual discrimination task consisted of a flashing LED presented at 24 Hz. The NoGo stimulus for the more difficult (“hard”) visual discrimination task consisted of a flashing LED presented at 12 Hz. Go and NoGo training were identical to the procedures described for the AM rate auditory discrimination task.

### Control (sham surgery) experiments

Control (sham surgery) SSCD animals (*n* = 4, easy auditory discrimination task; *n* = 4, easy visual discrimination task) were anesthetized with isoflurane (1.0%) and prepared for stereotaxic surgery. An incision was made over the nuchal muscles on the left side of the head just posterior to the ear. Once exposed the nuchal muscles were sharply and then bluntly dissected to expose the left superior bulla. A 5.0 mm opening was made with a 1.5 mm diamond bur. The intact superior (anterior) semicircular canal was directly visualized but was not fenestrated. The open bulla was then sealed/partitioned with Sterile Silastic (Dow Chemical Company, Midland, MI) to partition the air-filled bulla from the overlying neck muscles thereby restoring the normal air-filled middle ear and avoiding a true conductive hearing loss. Condensation on the interior surface of the Silastic seal was deemed indicative of this restoration of function. Finally, the reattached muscles were glued to the skull with Medbond tissue glue (Stoelting Co., Wood Dale, IL) which allowed c+VEMP testing after the control (sham surgery) procedure. The incision was closed with a running locked 4-0 Vicryl suture (Ethicon US, LLC, New Brunswick, NJ) and topical antibiotic was applied to the wound. Preoperative c+VEMPs and ABRs were performed and repeated at post day 15. Behavioral training for the easy auditory and visual tasks was completed as described above. The easy auditory discrimination task and visual discrimination task was carried out for the same timeline as the SSCD group (post day 6–15).

## Results

In this study we collected behavioral, ABR, and c+VEMP data from 33 male and female Mongolian gerbils. An auditory discrimination task and visual discrimination task was used, and each had two difficulty levels (easy auditory [*n* = 7], hard auditory [*n* = 8], easy visual [*n* = 11], hard visual [*n* = 7]). Of those animals included in this study, 85% (*n* = 28) had no head tilt or circling after surgical creation of the SSCD and 15% (*n* = 8) had head tilt that resolved by post day 3 and before electrophysiologic and behavioral testing beginning on post day 7.

In humans, mild cognitive impairments have been associated with third window syndrome including SSCD prior to surgical treatment. These impairments have been shown to improve after surgery repair. In our recent gerbil SSCD model publication we showed that the site of dehiscence was spontaneously resurfaced *via* osteoneogenesis in the weeks following fenestration which is accompanied by a peak in c+VEMP amplitude and ABR threshold shift at post day 7 that progressively returned towards baseline between post days 14 and 21 (22). The duration required for resurfacing was a function of fenestration size. Therefore, we tracked the relationship between behavioral performance, and physiological measures associated with SSCD diagnostic findings (ABRs, c+VEMPs), and recovery as the SSCD spontaneously resurfaced.

### SSCD induced cognitive impairments in an auditory and visual Go-NoGo task

**Figure 1:**
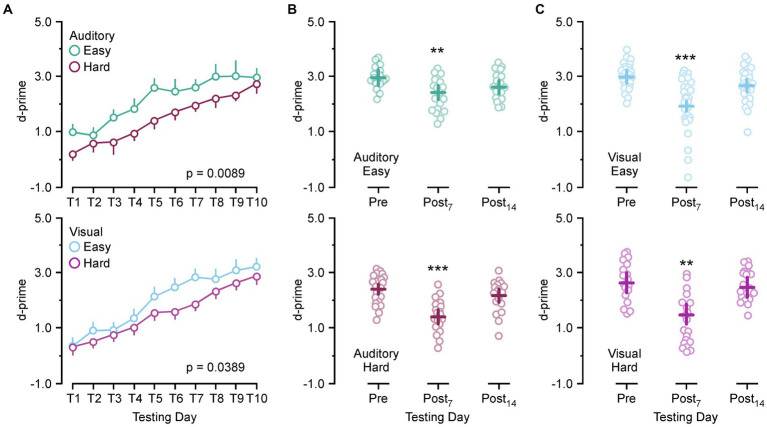
Behavioral impairments associated with postoperative days. **(A)** Line plot showing the group learning rates for the easy auditory discrimination task (green) and hard auditory discrimination task (purple) (top) and the easy visual discrimination task (blue) and hard visual discrimination task (purple) (bottom). **(B)** Scatter diagram showing comparisons between preoperative performance and postoperative day 7 (Postop_7_) and postoperative day 14 (Postop_14_) for the easy auditory discrimination task (green) (top) and hard auditory discrimination task (purple) (bottom). **(C)** Scatter diagram showing comparisons between preoperative performance and postoperative day 7 (Postop_7_) and postoperative day 14 (Postop_14_) for the easy visual discrimination task (blue) (top) and hard visual discrimination task (purple) (bottom). **p ≤ 0.001, ***p ≤ 0.0001.

Because there is a mild hearing impairment associated with SSCD we ran an auditory and visual version of the discrimination task to test for modality specific effects on behavioral performance. For each modality (auditory/visual) a hard and easy version of the task was used to identify if task difficulty was a factor in SSCD cognitive impairment. Figure 1 shows the learning curves for both tasks and the behavioral impairments associated with the peak of the ABR threshold shift and c+VEMP amplitude increase (post day 6, 7, 8) and the progressive shift towards baseline as the dehiscence undergoes resurfacing (post day 13, 14, 15). Figure 1A shows the learning curves over 10 days of behavioral testing for both versions and difficulty levels of the task. Criterion was set at a d-prime of 1.5, thus both the easy auditory discrimination task and easy visual discrimination task were learned at the same rate, with a similar delay in learning the more difficult task.

There was no significant difference between the learning rate for the easy auditory discrimination task and easy visual discrimination task [*F*(1,16) = 0.57, *p* = 0.45] or the hard auditory discrimination task and the hard visual discrimination task [*F*(1,13) = 0.16, *p* = 0.68]. Within each task there was a significant difference in the learning rate between the easy and hard task (auditory, [*F*(1,13) = 9.43, *p* = 0.0089]; [*F*(1,16) = 5.06, *p* = 0.0389]). After 10 days of the testing the animals were given surgical fenestrations of the superior (anterior) semicircular canal and allowed to recover for 5 days prior to being returned to the behavioral paradigm. The animals then received 10 more days of testing to create statistical comparisons at the peak of the physiological impairment (post day 7) and during the recovery process (post day 14). Comparing preoperative d-prime performance (training day 8–10) to post day 7 (post days 6–8) and post day 14 performance (post days 13–15) showed a significant main effect of SSCD on behavior [*F*(2,294) = 42.28, *p* < 0.0001] with significant decreases at post day 7 (preoperative 2.77 ± 0.085 vs. post day 7, 1.83 ± 0.074, [*p* < 0.0001]) and post day 14 (preoperative 2.77 ± 0.085 vs. post day 14, 2.5 ± 0.048, [*p* = 0.0431]). When compared by task difficulty the significant decrease at post day 7 remained (easy: preoperative 2.99 ± 0.095 vs. post day 7, 2.15 ± 0.093, [*p* < 0.0001]; hard, preoperative 2.50 ± 0.105 vs. post day 7, 1.44 ± 0.109, [*p* < 0.0001]), while the main effect at post 14 was no longer significant (easy: preoperative 2.99 ± 0.095 vs. post day 14, 2.68 ± 0.094, [*p* = 0.059]; hard, preoperative 2.50 ± 0.105 vs. post day 14, 2.32 ± 0.104, [*p* = 0.453]). We next divided the data by task modality and difficulty for individual comparisons. Figures 1A–D shows the behavioral impairment associated with SSCD for the easy auditory discrimination task, hard auditory discrimination task, easy visual discrimination task, and hard visual discrimination task. There were significant differences between the preoperative behavior and postop day 7 for both tasks and difficulties (easy auditory, preoperative 2.96 ± 0.115 vs. post day 7, 2.45 ± 0.131, [*p* = 0.0049]; hard auditory, preoperative 2.40 ± 0.108 vs. post day 7, 1.41 ± 0.103, [*p* < 0.0001]; easy visual, preoperative 3.01 ± 0.132 vs. post day 7, 1.96 ± 0.123, [*p* < 0.0001]; hard visual, preoperative 2.60 ± 0.190 vs. post day 7, 1.47 ± 0.181, [*p* = 0.0003]). By post day 14 these impairments had largely resolved (easy auditory, preoperative 2.96 ± 0.115 vs. post day 14, 2.63 ± 0.111, [*p* = 0.098]; hard auditory, preoperative 2.40 ± 0.108 vs. post day 14, 2.17 ± 0.103, [*p* = 0.297]; easy visual, preoperative 3.01 ± 0.132 vs. post day 14, 2.72 ± 0.133, [*p* = 0.274]; hard visual, preoperative 2.60 ± 0.190 vs. post day 14, 2.45 ± 0.32, [*p* = 0.831]).

### Behavioral metrics associated with cognitive impairments

**Figure 2:**
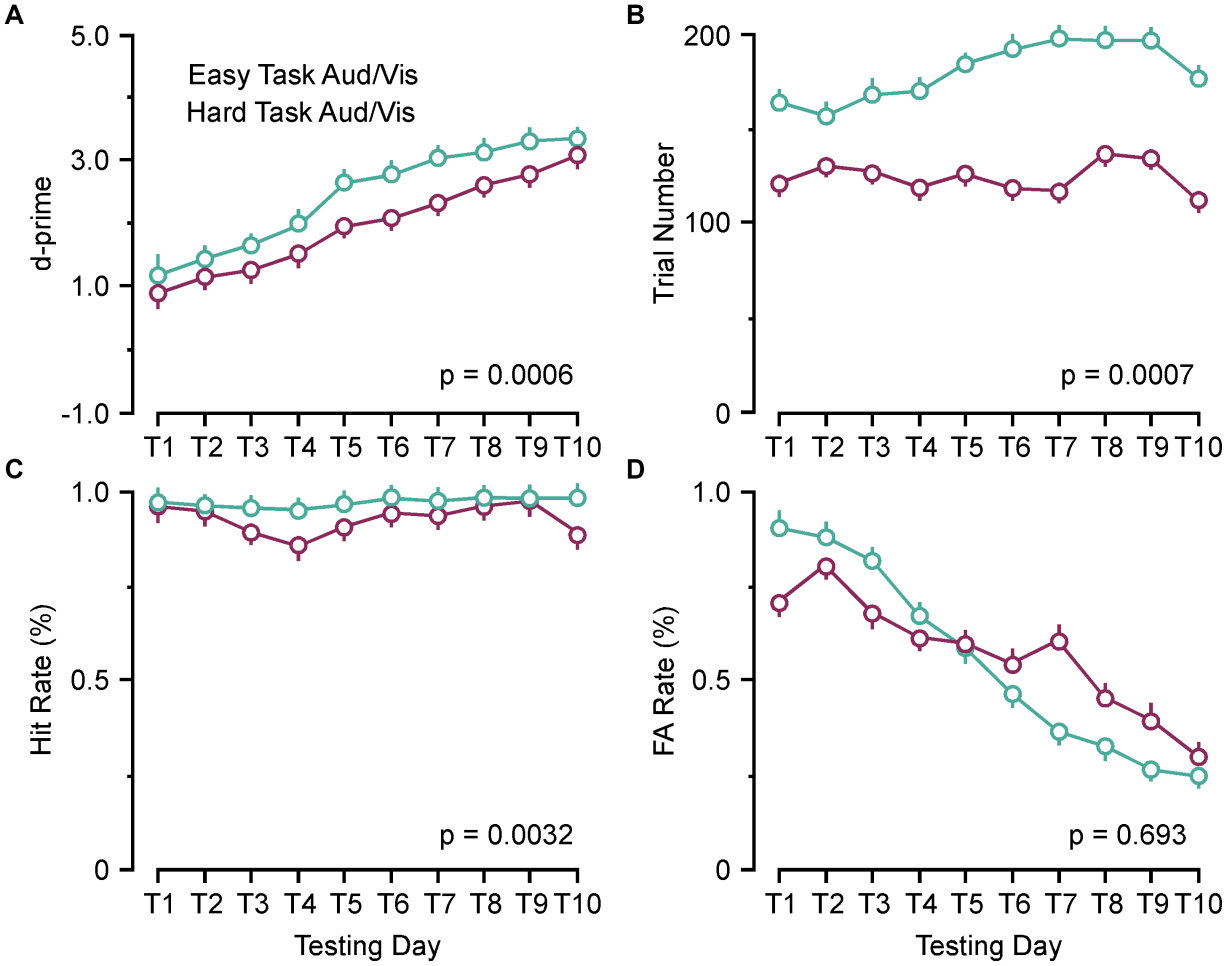
Behavioral parameters associated with performance (d-prime). **(A)** Line plots comparing the group learning rates for the pooled easy auditory and visual discrimination tasks (green) and hard auditory and visual discrimination (purple) tasks. **(B)** Line plot comparing the group trial numbers for the pooled easy auditory and visual discrimination tasks (green) and hard auditory and visual discrimination (purple) tasks. **(C)** Line plot comparing the group hit rates for the pooled easy auditory and visual discrimination tasks (green) and hard auditory and visual discrimination (purple) tasks. **(D)** Line plot comparing the group false alarm (FA) rates (%) for the pooled easy auditory and visual discrimination tasks (green) and hard auditory and visual discrimination (purple) tasks. Aud, auditory discrimination task; Vis, visual discrimination task.

As previously reported, there are individual variances associated with each animal concerning magnitude of the ABR threshold shifts and changes to c+VEMP amplitudes as the dehiscence is resurfaced *via* spontaneous osteoneogenesis. Therefore, we wanted to see if changes in ABR and c+VEMP properties were correlated with behavioral performance at post day 14. The behavioral measure d-prime is an expression of the ratio of the Hit rate to the FA rate. There are two main factors that can increase or decrease d-prime. Figure 2 shows the factors in this study that contributed to the impairments we report at post day 7 and the return towards terminal behavior scores at post day 14. Our results showed d-prime naturally increases for both the easy and hard task as the animals learn to respond correctly [*F*(1,35) = 14.72, *p* = 0.0006] (Figure 2A). A significantly smaller number of trials occurred in the harder task [*F*(1,35) = 14.85, *p* = 0.0005] (Figure 2B). Figure 2C shows that this leads to a smaller Hit rate in the harder task [*F*(1,35) = 10.05, *p* = 0.0032], but similarly decreasing FA rates [*F*(1,35) = 0.157, *p* = 0.693] (Figure 2D).

**Figure 3:**
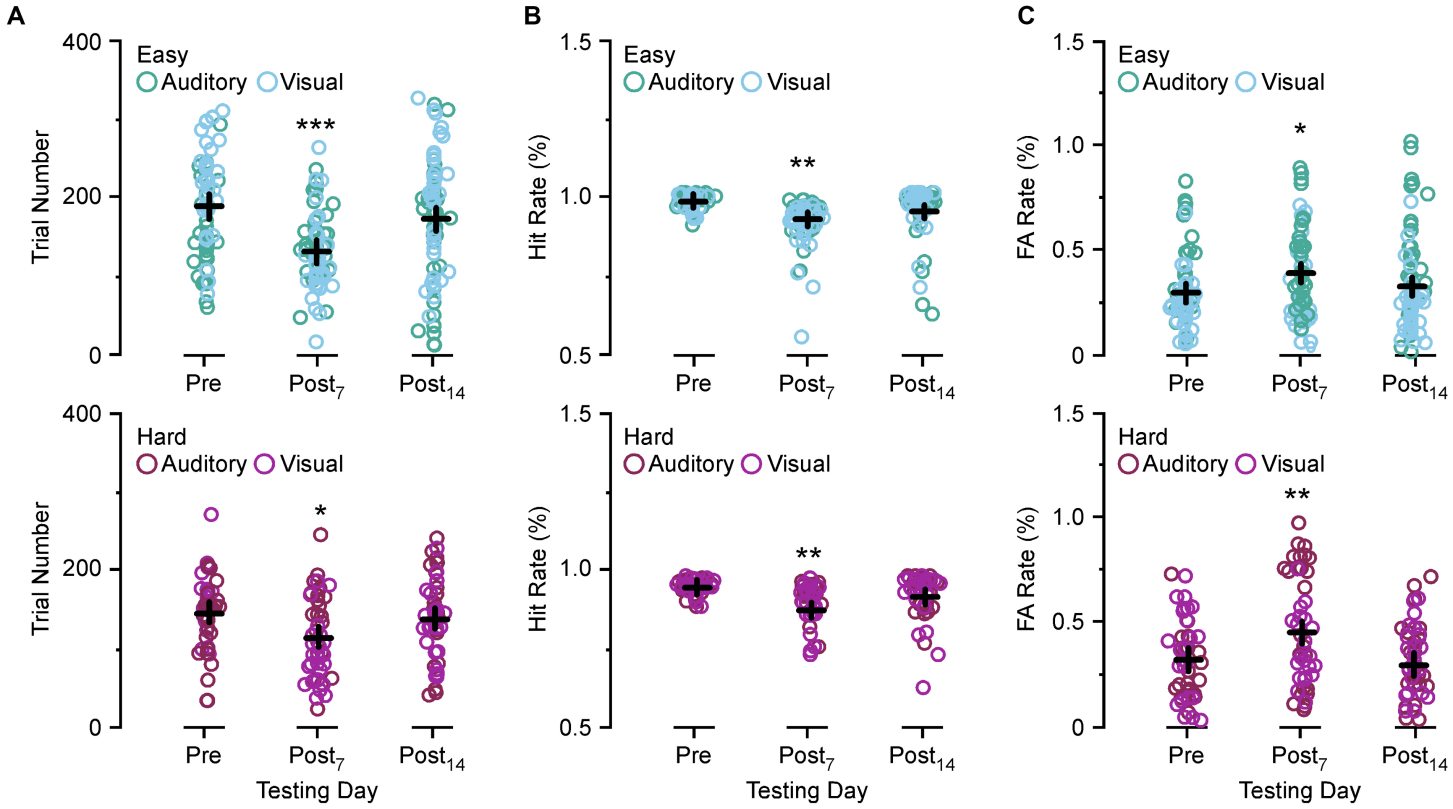
Comparison of the trial numbers performed, Hit rate, and false alarm (FA) rate between preoperative and postoperative days. **(A)** Scatter diagram showing group comparisons of average trial numbers performed between preoperative testing and postoperative day 7 and 14 for the easy auditory discrimination task (green) and easy visual discrimination task (blue) (top) and hard auditory discrimination task (red) and hard visual discrimination task (purple) (bottom). **(B)** Scatter diagram showing group comparisons of Hit rate between preoperative testing and postoperative day 7 (Postop_7_) and postoperative day 14 (Postop_14_) for the easy auditory discrimination task (green) and easy visual discrimination task (blue) (top) and hard visual discrimination task (purple) (bottom). **(C)** Scatter diagram showing group comparisons of average FA rate between preoperative testing and postoperative day 7 (Postop_7_) and postoperative day 14 (Postop_14_) for the easy auditory discrimination task (green) and easy visual discrimination task (blue) (top) and hard visual discrimination task (purple) (bottom). *p ≤ 0.01, **p ≤ 0.001, ***p ≤ 0.0001.

Figure 3 compares the number of trials performed, Hit rate, and FA rate between preoperative behavior, post day 7, and post day 14 for the easy and hard versions of the auditory discrimination task and the visual discrimination task. In Figure 3A both the easy and the hard auditory discrimination task and the visual discrimination task show a significant reduction in overall trial number at post day 7 (easy, preoperative 202.3 ± 8.2 vs. post day 7, 133.2 ± 7.2, [*p* < 0.0001]; hard, preoperative 136.8 ± 7.53 vs. post day 7, 105.5 ± 5.77, [*p* = 0.0109]) that returned towards baseline at post day 14 (easy, preoperative 202.3 ± 8.2 vs. post day 14, 173.8 ± 8.30, [*p* = 0.054]; hard, preoperative 136.8 ± 7.53 vs. post day 14, 128.8 ± 8.35, [*p* = 0.735]). The same effect is present for the Hit rate in Figure 3B (easy, preoperative 0.97 ± 0.012 vs. post day 7, 0.91 ± 0.075, [*p* = 0.0015]; hard, preoperative 0.96 ± 0.013 vs. post day 7, 0.89 ± 0.035, [*p* = 0.0007]; easy, preoperative 0.97 ± 0.012 vs. post day 14, 0.94 ± 0.011, [*p* = 0.126]; hard, preoperative 0.96 ± 0.013 vs. post day 14, 0.93 ± 0.031, [*p* = 0.258]), and FA rate in Figure 3C (easy, preoperative 0.27 ± 0.019 vs. post day 7, 0.37 ± 0.061, [*p* = 0.0303]; hard, preoperative 0.36 ± 0.031 vs. post day 7, 0.49 ± 0.091, [*p* = 0.0141]; easy, preoperative 0.27 ± 0.012 vs. post day 14, 0.30 ± 0.018, [*p* = 0.720]; hard, preoperative 0.36 ± 0.031 vs. post day 14, 0.33 ± 0.027, [*p* = 0.847]). Overall, the SSCD lowered the number of trials, decreased the Hit rate, and increased the FA rate, suggesting that there was not a single factor driving the impairment.

### SSCD induced physiological factors associated with cognitive impairments

**Figure 4:**
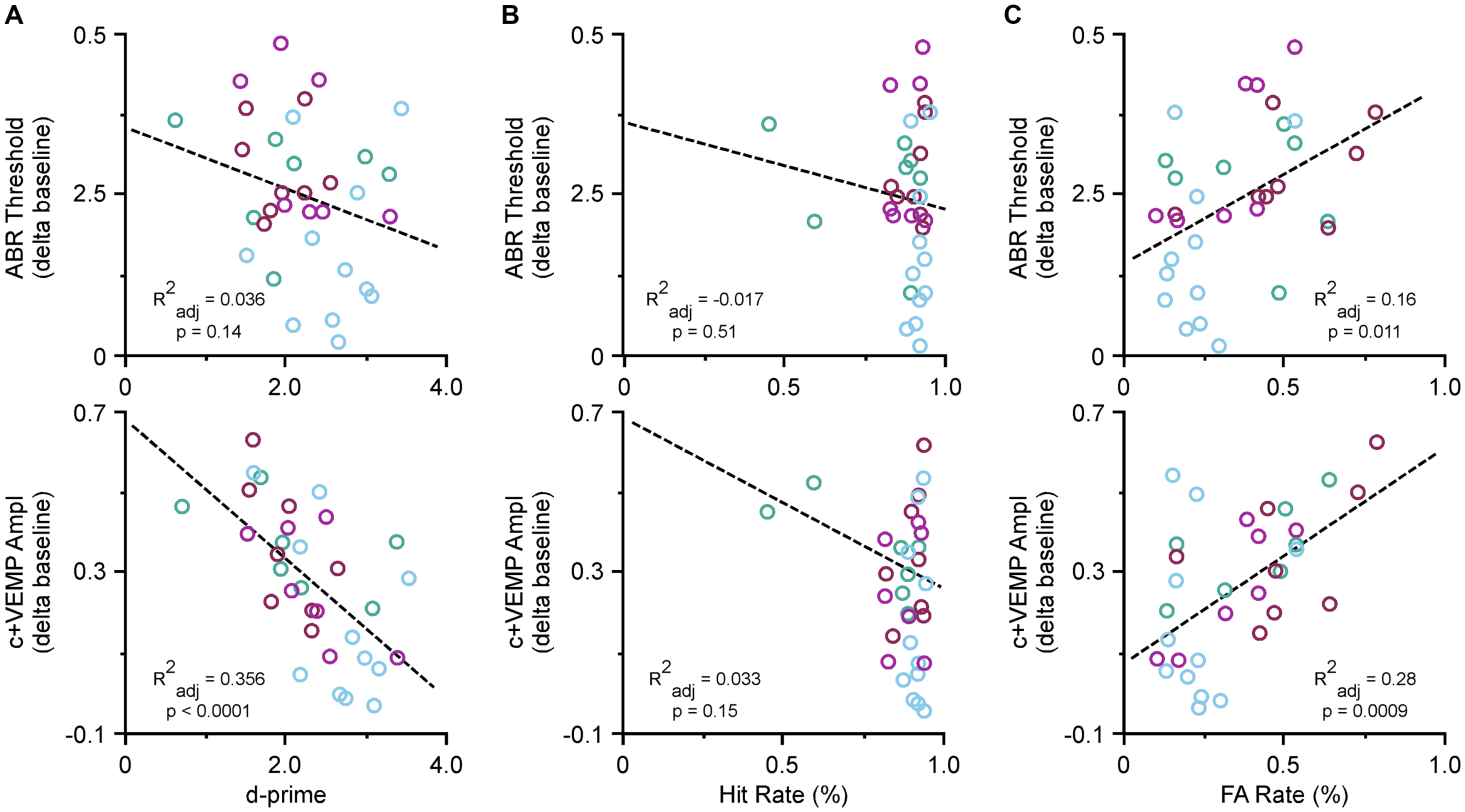
Correlations between ABR thresholds, c+VEMP amplitudes, d-primes, hit rates and false alarm (FA) rates. **(A)** Scatter plot showing correlations across all tasks for ABR thresholds and d-primes (easy auditory discrimination task [green] and easy visual discrimination task [blue], and hard auditory discrimination task [red] and hard visual discrimination task [purple]) (top) and c+VEMP amplitudes and d-primes (easy auditory discrimination task [green] and easy visual discrimination task [blue], and hard auditory discrimination task [red] and hard visual discrimination task [purple]) (bottom). **(B)** Scatter plot showing correlations across all tasks for ABR thresholds and hit rates (top) and c+VEMP amplitudes and hit rates (bottom). **(C)** Scatter plot showing correlations across all tasks for ABR thresholds and false alarm (FA) rates (%) (easy auditory discrimination task [green] and easy visual discrimination task [blue], and hard auditory discrimination task [red] and hard visual discrimination task [purple]) (top) and c+VEMP amplitudes and FA rates (easy auditory discrimination task [green] and easy visual discrimination task [blue], and hard auditory discrimination task [red] and hard visual discrimination task [purple]) (bottom).

Due to the recovery phase of the animals after SSCD and from being food deprived, we did not collect ABR or c+VEMP data at post day 7; however, we did take final recordings at post day 15. Therefore, we tested whether there were specific correlations between ABR thresholds, c+VEMP amplitudes, Hit rates, or FA rates and the last few days of behavioral performance. Figure 4 shows the correlations for the behavioral data at post day 14. Figure 4A illustrates the correlation between ABR thresholds (top) c+VEMP amplitudes (bottom) and d-prime for the auditory and visual discrimination tasks. There was not a significant effect of threshold shift on performance (ABR Threshold [dB] = 0.39–0.05 * behavior (d-prime), adjusted *R*^2^ = 0.03, *p* = 0.146); however, there was a significant correlation of c+VEMP amplitude on behavior (c+VEMP amplitude [μV] = 0.75–0.20 * behavior [d-prime], adjusted *R*^2^ = 0.35, *p* ≤ 0.0001). Despite not having a straightforward group deficit at post day 14, individual differences in animals showed that higher c+VEMP amplitudes at post day 14 were associated with lower performance. Figure 4B shows the correlation between ABR thresholds (top), c+VEMP amplitudes (bottom) and Hit rate for the auditory and visual tasks. It should be noted that Hit rate was not affected by either threshold shifts (ABR Threshold [dB] = 0.40–0.146 * Hit Rate [%], adjusted *R*^2^ = −0.017, *p* = 0.513) or c+VEMP amplitudes (c+VEMP amplitude [μV] = 0.75–0.52 * Hit rate (%), adjusted *R*^2^ = 0.033, *p* = 0.156). However, FA rate was significantly affected by both the threshold shift (ABR Threshold [dB] = 0.182 + 0.287 * FA rate (%), adjusted *R*^2^ = 0.16, *p* = 0.011) and the c+VEMP amplitudes (c+VEMP amplitude [μV] = 0.068 + 0.610 * FA rate (%), adjusted *R*^2^ = 0.28, *p* = 0.0009; Figure 4C). This suggests that interactions between vestibular dysfunction associated with increased c+VEMP amplitudes can lead to behavioral impairments by influencing FA rates.

### Control (sham surgery) experiments

**Figure 5:**
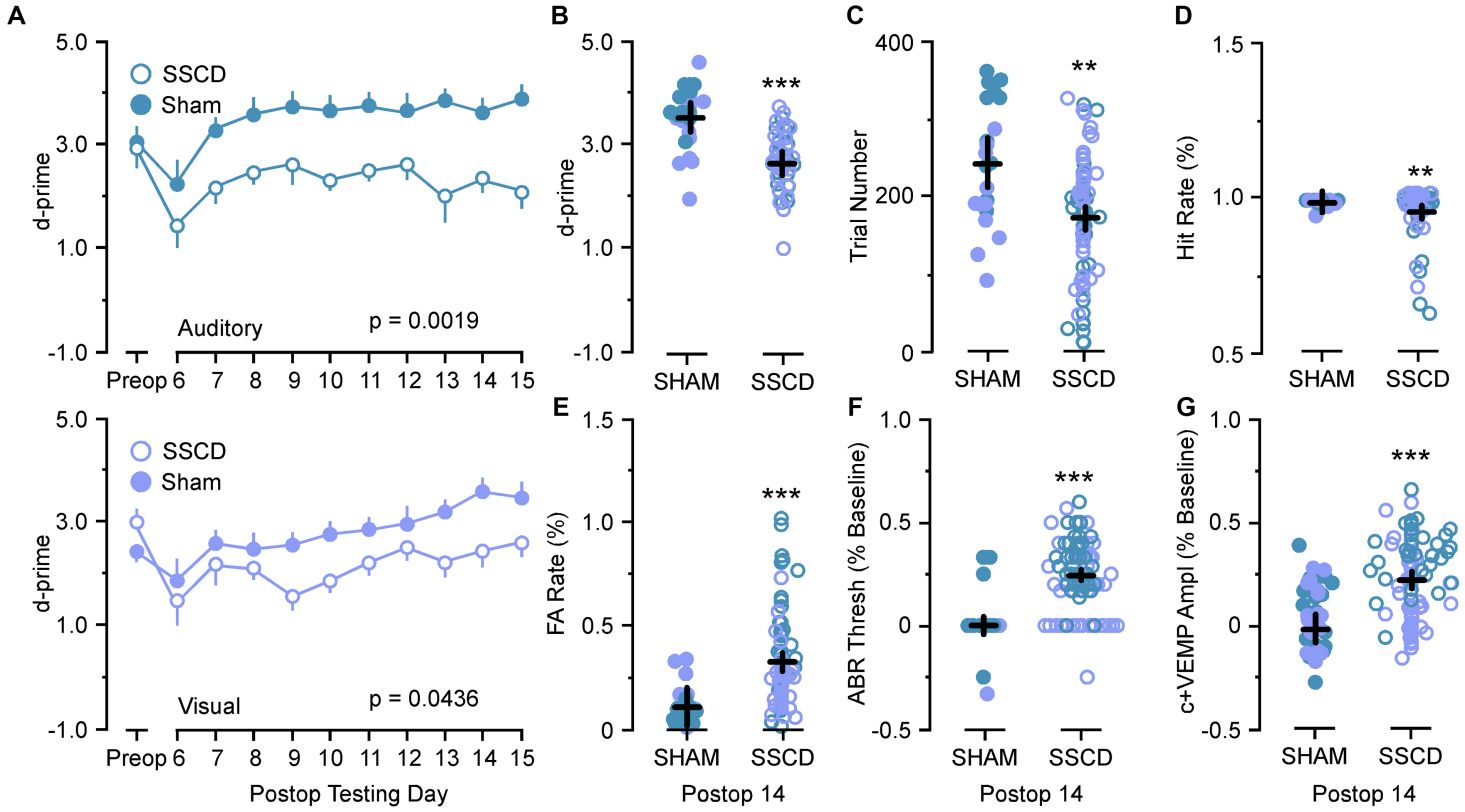
Statistical comparisons between SSCD and SSCD sham control animals. **(A)** Line graph comparing behavioral performance (d-prime) between SSCD (open blue) group and control (sham surgery) (solid blue) group for the easy auditory discrimination task across testing days (top). Line graph showing comparing behavioral performance (d-prime) between SSCD (open lavender) and control (sham surgery) (solid lavender) animals for the easy visual discrimination task across testing days (bottom). **(B)** Scatter plot showing the significant increase in d-primes in the control (sham surgery) group (solid colors) over the SSCD group (open colors) at postoperative day 14 (postoperative days 13–15) for both the easy auditory discrimination task (blue) and easy visual discrimination task (lavender). **(C)** Scatter plot showing that the control (sham surgery) group (solid colors) ran consistently more trials in both easy auditory discrimination task (blue) and easy visual discrimination task (lavender) compared to the SSCD group (open colors) at postoperative day 14. **(D)** Scatter plot showing the persistent slight decrease in Hit Rate for the SSCD group (open colors) compared to the control (sham surgery) group (solid colors) for both the easy auditory discrimination task (blue) and the easy visual discrimination task (lavender) at postoperative day 14. **(E)** Scatter plot showing the persistent elevation of false alarm (FA) rates on both the easy auditory discrimination task (blue) and the easy visual discrimination task (lavender) for the SSCD group (open colors) compared to the control (sham surgery) group (solid colors). **(F)** Scatter plot showing the persistent elevation of ABR thresholds for some SSCD animals (open colors) in both the easy auditory discrimination task (blue) and the easy visual discrimination task (lavender) at postoperative day 14 compared to the control (sham surgery) animals (solid colors). **(G)** Scatter plot showing the persistent elevation of c+VEMP amplitudes for some SSCD animals (open colors) in both the easy auditory discrimination task (blue) and the easy visual discrimination task (lavender) at postoperative day 14 compared to the control (sham surgery) animals (solid colors). ABR, auditory brainstem response; c+VEMP, positive cervical vestibular evoked myogenic potential; FA, false alarm; Postop, postoperative; Preop, preoperative; SHAM, control (sham surgery); SSCD, superior semicircular canal dehiscence. **p < 0.01, ***p < 0.001.

The results of the control (sham surgery) experiments are shown in Table 1; Figure 5. For these analyses we compared data from control (sham surgery) animals (*n* = 8) to SSCD animals at post day 7 (post days 6–8) and post day 14 (post days 13–15) that had completed 10 days of training followed by 10 days of postoperative training in the easy auditory task (*n* = 4) and the easy visual task (*n* = 4; Table 1; Figure 5). There was an additional learning phase in the sham group that led to a significant difference between the sham control and SSCD animals for both the easy discrimination auditory task [*F*(1,9) = 18.79, *p* = 0.0019] and the easy visual discrimination task [*F*(1,13) = 4.99, *p* = 0.0436]. This could suggest that even though the SSCD animals’ behavioral performance returns to preoperative levels, that the vestibular impairment might have delayed or possibly prevented the additional learning phase. This additional learning was more prominent in the easy auditory discrimination task compared to the easy visual discrimination task. Further evidence is presented in the statistical comparisons of the behavioral metrics and peripheral ear physiology at post day 7 and post day 14 (Table 1; Figure 5).

**Table 1:** Statistical comparisons between SSCD and sham controls for easy auditory task and easy visual task behavioral metrics and physiology.

	Easy auditory task post day 7	Easy auditory task post day 14	Easy visual task post day 7	Easy visual task post day 14
d-primes	*p* = 0.0060^*^	*p* < 0.0001^***^	*p* = 0.0452^*^	*p* = 0.018^*^
Trial number	*p* = 0.0007^**^	*p* ≤ 0.0001^***^	*p* = 0.617 n.s.	*p* = 0.613 n.s.
Hit rates	*p* = 0.489 n.s.	*p* = 0.021^*^	*p* = 0.0226^*^	*p* = 0.022^*^
False Alarm rates	*p* < 0.0001^***^	*p* = 0.0022^**^	*p* = 0.0182^*^	*p* = 0.0005^**^
ABR thresholds	NA	*p* < 0.0001^***^	NA	*p* < 0.0001^***^
c+VEMP amplitudes	NA	*p* < 0.0001^***^	NA	*p* < 0.0001^***^

*p < 0.05; **p < 0.01; ***p < 0.001. ABR, auditory brainstem response; c+VEMP, positive cervical vestibular evoked myogenic potential; NA, not applicable; n.s., not statistically significant; Post, postoperative.

For the animals that were run through the easy auditory discrimination task there were highly significant differences in the behavioral metrics at post day 7 and post day 14 when compared to SSCD animals. Sham animals ran significantly more trials at both post day 7 (sham post day 7, 208 ± 15 vs. SSCD post day 7, 143 ± 9 [*p* = 0.0007]) and post day 14 (sham post day 14, 307 ± 24 vs. SSCD post day 14, 155 ± 15 [*p* < 0.0001]). Their d-primes were significantly higher as well at both time points (sham post day 7, 3.04 ± 0.18 vs. SSCD post day 7, 2.42 ± 0.11 [*p* = 0.0060]; sham post day 14, 3.79 ± 0.21 vs. SSCD post day 14, 2.31 ± 0.13 [*p* < 0.0001]). This was driven by significant differences in the FA rates (sham post day 7, 13.3 ± 0.04 vs. SSCD post day 7, 37.8 ± 0.03 [*p* < 0.0001]; sham post day 14, 7.6 ± 0.19 vs. SSCD post day 14, 22.3 ± 0.02 [*p* = 0.0022]) as opposed to the differences in Hit rates (sham post day 7, 94.4 ± 0.04 vs. SSCD post day 7, 91.1 ± 0.02 [*p* = 0.489]; sham post day 14, 91.99 ± 0.008 vs. SSCD post day 14, 96.5 ± 0.005 [*p* = 0.021]), which were only different at post day 14.

Performance in the easy visual discrimination task was also better in the sham group; however, the results were not as robust. These sham animals did not run more trials than the SSCD animals at either post day 7 (sham post day 7, 132 ± 17 vs. SSCD post day 7, 122 ± 10 [*p* = 0.617]) or post day 14 (sham post day 14, 192 ± 13 vs. SSCD post day 14, 178 ± 22 [*p* = 0.613]). There were slightly significant increases in d-primes at post day 7 (sham post day 7, 2.43 ± 0.25 vs. SSCD post day 7, 1.96 ± 0.15 [*p* = 0.0452]) and post day 14 (sham post day 14, 3.32 ± 0.20 vs. SSCD post day 14, 2.72 ± 0.12 [*p* = 0.018]), but not nearly as robustly significant as the easy auditory task sham animal d-primes. This was accompanied by slightly significant increases in Hit rate (sham post day 7, 97.3 ± 0.004 vs. SSCD post day 7, 95.2 ± 0.007 [*p* = 0.0226]; sham post day 14, 98.01 ± 0.03 vs. SSCD post day 14, 87.9 ± 0.02 [*p* = 0.022]) and lower FA rates (sham post day 7, 23.9 ± 0.02 vs. SSCD post day 7, 37.3 ± 0.04 [*p* = 0.0182]; sham post day 14, 14.7 ± 0.07 vs. SSCD post day 14, 49.0 ± 0.04 [*p* = 0.0005]) at both postoperative points.

Finally, significant increases in ABR thresholds remained in the SSCD group for both tasks, whereas sham animals showed no real changes from baseline at post day 14 (easy auditory discrimination task; sham post day 14, 3.3 ± 0.02 vs. SSCD post day 14, 31.0 ± 0.01 [*p* < 0.0001]; easy visual discrimination task; sham post day 14, 1.1 ± 0.03 vs. SSCD post day 14, 19.3 ± 0.02 [*p* < 0.0001]). The same trend was present for c+VEMP amplitude measurements (easy auditory task; sham post day 14, 1.1 ± 0.03 vs. SSCD post day 14, 19.3 ± 0.02 [*p* < 0.0001]; easy visual task; sham post day 14, −1.9 ± 0.05 vs. SSCD post day 14, 12.9 ± 0.03 [*p* < 0.0001]). As expected, this suggests that the sham surgery does not impose a change to cochlear or vestibular function.

## Discussion

Vestibular function plays a crucial role in decision-making, as it provides ongoing real time feedback about body position, balance, and motion that integrates with auditory, visual, and somatosensory inputs (23–27) to make precise judgements regarding our orientation, velocity, and acceleration in relation to our head/world position in space (28). Together the integration of vestibular information across the central nervous system culminates in the emergence of cognitive processing (29). Conversely, dysfunction of the vestibular system leads to cognitive impairments (30). In this study we used a form of vestibular injury, i.e., SSCD, to ask how decision-making in an associative conditioning task is affected by aberrant asymmetric vestibular output. We found decreases in decision-making performance that were highly correlated with the peak and return towards baseline of ABR and c+VEMP measurements that we previously reported (22) and observed in the current study. The c+VEMP amplitude was specifically and highly correlated with variable recovery and lingering decision-making performance deficits. These were consistent across both a visual and auditory task of varying difficulty.

### Human cognitive impairment in SSCD and third window syndrome

Previously, there has been controversy over whether there is a direct link between vestibular disorders and certain forms of cognitive impairment (31). However, recent research has shown that the relationship between vestibular function and cognition is incredibly intricate (32, 33), with a high incidence of some form of impairment present in patients with third window syndrome (13, 17, 34–44). In this context, the term “cognitive dysfunction” is being used broadly to refer to characteristics that are not directly influenced by vestibular sensorimotor coupling.

In patients with third window syndrome, including SSCD, cognitive dysfunction is almost always present due to otolithic asymmetry. This is not typically seen in disorders such as benign positional vertigo, vestibular neuronitis, or other rotational receptor dysfunctions (45). Patients with third window syndrome often report cognitive difficulties such as poor memory and concentration, trouble reading, forgetting words, and difficulty expressing themselves. To understand the intricate relationship between vestibular dysfunction and cognitive impairment, we must examine behavioral and anatomical studies in animals. Hitier et al. provide an excellent review of the neuroanatomical pathways from the vestibule to the central nervous system in rodents, cats, and non-human primates (29). Hitier et al. described five major pathways that integrate vestibular information throughout the brain, each with a specific function related to spatial learning and memory (29). These include: “(1) a vestibulo-thalamic-cortical pathway for environmental spatial integration, (2) a tegmental-thalamic-entorhinal pathway for calculating head direction, (3) a reticularis pontis oralis-supramammillary-septal pathway to the hippocampus involved with spatial memory and object recognition, (4) a cerebellar-thalamic-cortical pathway that supports spatial learning, and (5) a vestibular-thalamic-striatal pathway that supports spatial learning and memory.” The detailed anatomical pathways are beyond the scope of this discussion, but it is clear that vestibular dysfunctions, such as those seen in third window syndrome, will affect normal activity along these pathways, resulting in cognitive impairments. The complexity of the vestibular system’s non-classical sensory function has led to debates about the relationship between vestibular function and cognition. In contrast to classic sensory systems like vision, which have modal-specific inputs with straightforward pathways to the cortex *via* the thalamus, the vestibular system’s sensory inputs are more complex. The brain regions contain stable receptive fields where sensory stimuli are represented by external maps. These maps are retinotopic, tonotopic, and somatotopic, representing the peripheral receptors of the retina, cochlea, and skin throughout each modality specific neuraxis. Vestibular pathways heavily integrate with these modalities through direct vestibular nucleus projections and multisynaptic pathways to higher-order brain regions in the midbrain and thalamus. The core regions integrate environmental spatial auditory and visual information, proprioceptive somatotopic information, and vestibular information about head direction, angular velocity, and momentum. Vestibular information is continually updated and lacks a classical central topographical map, making it difficult to interpret and study. Although animal research investigating this topic is limited, human data offer interesting clues as to how vestibular dysfunction can cause cognitive impairment.

### Behavioral features associated with vestibular function/dysfunction and cognitive impairment

To ask questions about cognition in lower animals such as rodents we turn to behavioral paradigms. Many previous animal studies have used unilateral labyrinthectomy and other forms of vestibular stimulation to probe the effect of vestibular dysfunction on various behaviors. The effects of vestibular manipulation on simple behaviors such as open field exploration suggest a definite role is spatial exploratory behavior (46, 47), while spatial navigation in the Morris water maze requires vestibular dependent cues (48). Reference memory in a radial arm maze task is significantly impaired by loss of vestibular information (49–51). Vestibular information even seems to be important for non-spatial processes such as object recognition (52). Studies using non-spatial associative conditioning have not been previously used to examine the effect of vestibular dysfunction on decision-making despite large input to the associative conditioning centers of the striatum (53–55). In this study we used a behavioral task with a decision-making component to ask whether superior semicircular canal dehiscence will produce measurable performance deficits. We found that at a postoperative timepoint associated with the peak of the auditory and vestibular physiological disruption (22), 7 days after SSCD, showed significant behavioral impairments to decision-making. Hit rates were lowered, misses were increased, FAs increased, and correct rejections decreased, leading to overall lowering of d-prime performance measures. These results were consistent across task modality (auditory/visual) and difficulty (easy/hard) suggesting a conserved source for the impairment. It further suggests that the raised auditory thresholds in the left ear were not the causative factor in the auditory task impairments. Observation of the animals showed that they were often confused and would go to an adjacent non-used food trough (misses) or would pause and then continue to the correct food trough (FA, NoGo trial). The increased FA rate was specifically correlated with the presence of a lingering increase in c+VEMP amplitude, which were also significantly correlated with lowered d-primes. Animals that had more recovery towards baseline, had lower c+VEMP amplitudes compared to baseline, lower FA rates, and thus higher d-primes. This suggests a clear connection between the decision-making errors which result in false alarms and the asymmetric vestibular dysfunction. This associative conditioning task has cortical dependent properties to it (56) that could suggest that errors in the decision-making could be due to cortical processing errors that are passed down to the striatum. There could also be hippocampal dependent components associated with spatial reference and navigation to the correct food trough, of which vestibular dysfunction disrupts the allocentric and egocentric reference required to navigate the behavioral arena (49, 50). Along these lines, there could be cerebellar aspects to the impairments, as this structure governs features of vestibular mediated spatial navigation (57).

### Control (sham surgery) experiments

There was an additional learning phase in the control (sham surgery) group that led to a significant difference between the control (sham surgery) and SSCD groups for both the easy auditory discrimination task and the easy visual discrimination task. This could suggest that even though the SSCD animals’ behavioral performance returns to preoperative levels, the vestibular impairment might have delayed or possibly prevented the additional learning phase. This additional learning was more prominent in the easy auditory discrimination task compared to the easy visual discrimination task. Further evidence was presented in the statistical comparisons of the behavioral metrics at post day 7 and post day 14 and the peripheral ear physiology at post day 14 (Table 1; Figure 5). For the control (sham surgery) group that completed the easy auditory discrimination task there were highly significant differences in the behavioral metrics at post day 7 and post day 14 when compared to the SSCD group. Their d-primes were significantly higher as well at both time points (Table 1). This was driven by significant differences in the FA rates as opposed to the differences in Hit rates, which were only different at post day 14. Performance in the easy visual discrimination task was also better in the sham group; however, the results were not as robust. Finally, despite the lack of significance when comparing preoperative to post 14 ABR thresholds, and c+VEMPs in the SSCD animals as a group, the comparisons to control (sham surgery) animals revealed that peripheral physiology was still recovering in the SSCD group.

### Gerbils as an ideal model to study SSCD central impairments

Altogether, this model of SSCD induced impairment in gerbils adds a powerful new scientific tool to study the effects of vestibular dysfunction on central circuits involved with learning, memory, behavior, and cognition. There is extensive literature that characterizes gerbil visual and auditory function. The gerbil is a diurnal rodent possessing a retina and cone density that is more similar to humans than those of mice and rats (58), providing excellent motion and depth perception (58–60). For auditory function, gerbils possess robust low frequency hearing, and its audiogram overlaps with the human audiogram (61). This contrasts with more standard rodent models, such as mice and rats, as they possess greater sensitivity over very high frequencies and are limited at low frequencies (62, 63). The gerbil also possesses robust auditory perceptual skill (56, 64–81) that is vulnerable to hearing impairment (71, 75, 76, 82–85). Furthermore, previous work in gerbils also demonstrated that cognitive variables, such as the ability to generalize a learned rule, are vulnerable to hearing loss (86). More recently, the gerbil has been utilized as a model to assess temporal integration, a hallmark of cognitive function, during auditory decision-making (80, 84, 87). Thus, the gerbil is a suitable animal model for assessing cognitive function, particularly across visual and auditory domains.

An important issue with this animal model, in the context of measuring cognitive dysfunction/impaired decision-making, is the question of when the acute vestibular injury induced by surgically creating the SSCD becomes a chronic intermittent vestibular asymmetry. With acquired SSCD in patients, at some point the dehiscence is an acute change and because of the longstanding presence of the dehiscence, patients experience a chronic intermittent vestibular asymmetry. While the timeline for transition from acute injury to chronic condition in our gerbil model that parallels the human experience is unknown, in general rodent timelines are more rapid than in humans, as is the development milestones, aging and shorter life expectancy. We do not know the how the gerbil development timeline maps to that of humans; however, we do know that in adult rats, every day of the animal is approximately equivalent to 34.8 human days (88). It is known that in gerbils with unilateral labyrinthectomy (a more severe acute vestibular injury), vestibular compensation with vision shows improved vestibulo-ocular reflex (VOR) just 24 h after labyrinthectomy (89). Thus, we expect that any acute vestibular injury had resolved by post day 7. To minimize effects due to acute vestibular injury, our exclusion criteria included removing animals from the protocol that had persistent circling or head tilt present at post SSCD day 3. Of those animals included in this study (*n* = 33), 85% (*n* = 28) had no head tilt or circling after surgical creation of the SSCD and 15% (*n* = 8) had head tilt that resolved by post day 3 and before electrophysiologic and behavioral testing beginning on post day 7. We know from previous studies that the surgically created SSCD resurfaces *via* spontaneous osteoneogenesis by post day 14 (22), and our electrophysiologic and behavioral results in this study suggest that the SSCD also closed by post day 14.

To enhance our comprehension of the effects of the vestibular system on behavior, cognition, and symptomatology in central brain processes, it is essential to systematically investigate analogous animal models of vestibular dysfunction. This approach will allow us to create experiments that reproduce the peripheral causes of vestibular dysfunction and explore central alterations along the five pathways outlined earlier (29). Recently, our team has developed a gerbil model of third window syndrome that will serve as a foundation for a thorough examination of the symptomatology patients with SSCD experience (22). This model involves creating a fenestration in the superior semicircular canal that produces an inner ear/otic capsule-conductive hearing loss and sound-evoked changes in vestibular evoked myogenic potentials (c+VEMPs) similar to what is seen in humans with third window syndrome due to SSCD. As reported herein, this model also displays substantial reversible impairments in decision-making, which we can use to investigate the maladaptive central plasticity resulting from vestibular injury that leads to cognitive dysfunction. This eliminates the need for a second surgical intervention to plug the SSCD, offering us an experimental window to carry out electrophysiological and behavioral studies to evaluate decision-making as a proxy for cognitive dysfunction. This model will also aid in determining the precise vestibular contributions of the five primary neuroanatomical pathways described earlier (29) and assist in understanding the central plasticity resulting from SSCD. Advanced tools such as adeno-associated viruses (e.g., optogenetics; ChR2, DREADDs; HM4Di), along with improvements in awake behaving neurophysiology and *in vitro* whole-cell recording, can be employed to isolate and manipulate specific brain circuits selectively. This will allow us to pose highly informative questions about the effect of vestibular function on physiology (e.g., balance), emotional states (e.g., anxiety, fear), and cognitive-behavioral processes. By unraveling the intricacies of vestibular influence on central brain function, we should obtain new insights into the etiology of symptomatology in humans that will hopefully result in new treatment approaches for chronic third window syndrome symptoms and other vestibular-related conditions in the years ahead.

## Conclusion

Vestibular dysfunction in humans is associated with cognitive deficits that can lead to greatly reduced quality of life. Fortunately, treatments do exist for disorders such as SSCD, that involve surgical plugging or resurfacing the site of dehiscence. To enhance our understanding of how vestibular function affects behavior, cognition, and the central brain processes, it is necessary to systematically investigate vestibular disorders in animal models that are analogous to humans. Unlike humans who require surgical intervention, a particularly novel aspect of this SSCD model is the fenestration self-closure of the surgically created SSCD through spontaneous osteoneogenesis (22). This allows us the unique opportunity to study both impairment and recovery. We can also exploit this feature of the model to determine if there are persistent changes to central plasticity after recovery from injury. Herein, we report for the first-time, higher order associative decision-making impairments related to aberrant asymmetric vestibular output in an animal model of SSCD. These impairments resolve as the bone resurfaces *via* spontaneous osteoneogenesis; providing a unique window to study decision-making errors and their resolution. We can exploit this timeframe to design experiments that replicate the peripheral causes of vestibular dysfunction in SSCD and examine the central changes that underlie this dysfunction along many different brain circuits that receive vestibular inputs. We hope to expand these results in future experiments that study many of the circuits along which auditory, visual, and vestibular pathways integrate to drive learning, behavior, and higher-order cognition.

## Data availability statement

The raw data supporting the conclusions of this article will be made available by the authors, without undue reservation.

## Ethics statement

The animal study was approved by the Rutgers University IACUC reviewed and approved this research protocol (PROTO202000179). The study was conducted in accordance with the local legislation and institutional requirements.

## Author contributions

TMM: Conceptualization, Data curation, Formal analysis, Funding acquisition, Investigation, Methodology, Project administration, Resources, Software, Supervision, Validation, Visualization, Writing – original draft, Writing – review & editing. PAW: Conceptualization, Data curation, Formal analysis, Funding acquisition, Investigation, Methodology, Project administration, Resources, Software, Supervision, Validation, Visualization, Writing – original draft, Writing – review & editing. JN: Data curation, Formal analysis, Writing – review & editing. ED: Data curation, Formal analysis, Writing – review & editing. RDS: Data curation, Formal analysis, Writing – review & editing. AT: Data curation, Formal analysis, Writing – review & editing. NLC: Data curation, Formal analysis, Writing – review & editing. MAB: Data curation, Formal analysis, Writing – review & editing. SSH: Writing – review & editing, Data curation, Formal analysis. JDY: Formal analysis, Validation, Writing – original draft, Writing – review & editing.
